# Neurohormonal Regulation of Appetite and its Relationship with Stress: A Mini Literature Review

**DOI:** 10.7759/cureus.3032

**Published:** 2018-07-23

**Authors:** Armghan H Ans, Ibrar Anjum, Vaibhav Satija, Awaisha Inayat, Zain Asghar, Imran Akram, Bishesh Shrestha

**Affiliations:** 1 Cardiology, University of Pennsylvania, Philadelphia, USA; 2 Internal Medicine, The University of Texas MD Anderson Cancer Center, Houston, USA; 3 Internal Medicine, Saint Vincent Hospital, Worcester, USA; 4 Psychology, University of Karachi, Karachi, PAK; 5 Internal Medicine, Services Institute of Medical Sciences, Lahore, PAK; 6 Internal Medicine, King Edward Medical University/Mayo Hospital, Lahore, PAK; 7 Medicine, Bassett Medical Center, Cooperstown, USA

**Keywords:** stress, eating, obesity, appetite

## Abstract

Stress has long been known to affect eating behaviors in humans. Stress-induced hyperphagia is considered a potential cause for the development of obesity. Given the high prevalence of obesity and its association with other cardiovascular and metabolic disorders, the subject of stress-induced eating has become even more important. We reviewed data from past studies to further elucidate the relationship between stress, appetite regulation and eating patterns in humans. Even though it is difficult to say with certainty that a person exposed to stress will undereat or overeat, but certain assumptions can be made. Generally, acute stress results in decreased eating whereas chronic stress results in increased eating. Glucocorticoids, the effector molecules of the stress response, increase the tendency to consume high-calorie, palatable foods. Further studies that can link the biological markers of stress-response with the hormones and neurotransmitters of appetite regulation can broaden our understanding of the subject. These studies can provide a groundwork for the development of effective anti-obesity strategies.

## Introduction and background

Stress can be defined as an unpleasant, non-specific arousal state that challenges the body’s ability to maintain homeostasis [1]. Stress results in specific physiological, biochemical and behavioral changes in the body [2]. These changes aim to counteract the effects of stress. Different individuals have different responses to stress, and these responses vary according to the individual’s physiological, psychological and environmental factors [3]. Stress can be of various types, for example, physical (trauma, surgery), physiological (heavy exercise, pain), emotional (anxiety, fear) or social (interpersonal conflicts). Acute stress has temporary and immediate effects whereas chronic stress is persistent over a more extended period.

The relationship between stress and eating has long been a subject of discussion. Results from previous studies have shown that stress can affect appetite and eating behaviors of a person [4]. Also, the level of stressor has shown to have an impact on an individual’s eating habits.

The design of this study is systematic, and it builds upon itself. First, we will discuss the physiological regulation of appetite, with a primary focus on the mechanisms that can be influenced by stress. Then we will discuss how stress can affect these mechanisms and thus alter appetite. Finally, we will discuss different eating patterns seen under stress due to these alterations.

## Review

Appetite is a tightly regulated phenomenon and involves the interplay of various hormones and neurotransmitters for its effective control. These hormones are released from various parts of the body and affect appetite by modulating different factors including hunger, satiety and gut mobility. Two primary mechanisms by which appetite is regulated include central nervous system regulators and peripheral regulators. Hypothalamus has vital importance in the central regulation of the appetite [5]. Arcuate nucleus in hypothalamus produces two kinds of neurotransmitters: neuropeptide Y (NPY) and agouti-related peptide (AgRP) increase the food intake whereas alpha-melanocyte stimulating hormone (α-MSH) also known as pro-opiomelanocortin (POMC), and cocaine-and-amphetamine-regulated transcript (CART) decrease the food intake [6]. Cells are producing both kinds of neurotransmitters project to paraventricular nucleus; another nucleus in the hypothalamus. The paraventricular nucleus can modulate energy expenditure of body and food intake according to the input it receives from the arcuate nucleus [6]. Paraventricular nucleus also produces corticotropin-releasing hormone (CRH) which has shown to decrease appetite in humans [7]. The hypothalamus as a whole can receive input from higher brain centers, brain stem, and the bloodstream directly [8].

Leptin is a hormone synthesized in adipose tissue, and its serum concentrations are proportional to the amount of adipose tissue in the body [9]. It is secreted in the bloodstream from where it reaches the hypothalamus. In the hypothalamus, leptin downregulates the expression of orexigenic neurotransmitters (NPY/AgRP) and upregulates the expression of anorectic neurotransmitters (POMC/CART) [10]. The net result is decreased food intake and increased energy expenditure by the body. Leptin resistance has been shown to be associated with obesity in humans, and obese individuals have higher levels of leptin as compared to healthy individuals [11]. Additionally, leptin increases the release of a CRH from the hypothalamus [12] which in turn decreases appetite. Leptin and neuropeptide Y can be potential targets for future anti-obesity medications.

Peripheral factors constitute the other mechanism for appetite regulation. Cholecystokinin (CCK) is a peptide synthesized in the gut and brain and can be considered as a physiological ‘satiety factor’ [13]. Its primary function is to decrease food intake, but it also inhibits gastric emptying, promotes pancreatic secretions and causes gallbladder contractions [14]. CCK has two kinds of receptors: CCK-1 receptors (formerly known as CCK-A) and CCK-2 receptors (formerly known as CCK-B) [15]. CCK-1 receptors are located on the afferent vagal fibers in the body of the stomach and pyloric sphincter of the stomach. Upon activation, these vagal afferents can relay information to the brain resulting in inhibition of food intake [16]. Contraction of pyloric sphincter causes stomach distension which contributes to decreased food intake by activation of afferent vagal fibers and relaying information to the brain [17]. Furthermore, there is evidence that cholecystokinin may cause the release of CRH in the hypothalamus which contributes to a decrease in appetite [18].

Ghrelin, a peptide produced mainly in the mucosa of gastric fundus, is an appetite stimulant and can be considered as a signal to initiate meal as its levels rise preprandial and fall postprandially [19]. The fall in the levels of ghrelin postprandially appears to be nutrient specific, and carbohydrates play the most important role [20]. Ghrelin increases the appetite and induces a positive energy balance by increasing the level of orexigenic neurotransmitters (NPY/AgRP) in the arcuate nucleus of the hypothalamus [21]. Ghrelin levels are inversely correlated with adipose tissue mass in humans; being low in obesity and being high in conditions with weight loss, for example, anorexia nervosa [22].

Peptide YY (PYY) is a peptide belonging to the pancreatic polypeptide family and is released from small and large intestine after dietary intake [23]. Fat in the diet results in increased release of PYY as compared to carbohydrates and proteins [23]. Administration of PYY results in delayed gastric emptying, delayed gastric and pancreatic secretions, and increased water and electrolytes absorption from the ileum [24]. PYY has two forms in circulation: PYY 1-36 and PYY 3-36. PYY 3-36 is peripherally active form, crosses the blood-brain barrier, inhibits NPY neurons and decreases their expression in the hypothalamus leading to its anorectic effect [25]. These findings suggest the role of PYY as a satiety factor.

Studies have demonstrated that stress can bring changes in the hormones that are responsible for appetite regulation. This explains the different eating patterns of a person when exposed to stress. Acute stress typically results in ‘active fight-and-flight response’ with activation of the sympathetic-adrenal-medullary system and consequent release of catecholamines (adrenaline & noradrenaline) [26]. Noradrenaline has shown to suppress appetite during acute stress [27]. Other actions of catecholamines include a rise in blood pressure, increase in heart rate and decreased blood flow to the digestive system, kidneys, and skin [26].

On the other hand, chronic stress results in hyperactivation of hypothalamic-pituitary-adrenal (HPA) axis with resultant CRH, adrenocorticotropic hormone (ACTH) and glucocorticoids (cortisol) production [28]. CRH is a potent anorectic substance and has shown to influence food intake and energy balance both in humans and animals. In humans, CRH is believed to exert its anorectic effect by decreasing NPY synthesis and its release [29]. CRH also inhibits NPY-induced increased food intake [30]. Satiety hormone leptin exerts its anorectic effect partly by increasing the expression of CRH in the hypothalamus as discussed above. On the other hand, CRH results in the release of cortisol downstream from adrenals. Cortisol has shown to increase appetite as discussed below so that CRH can work as orexigenic substance as well.

In the hypothalamus, POMC gives rise to two peptides: α-MSH and ACTH [31]. Studies have shown that endogenous ACTH in the paraventricular nucleus of the hypothalamus along with α-MSH acts to decrease food intake [32]. Some studies indicate that acute stress can activate POMC neurons in the hypothalamus [33]. This shows the possible role of melanocortins along with ACTH in stress-induced anorexia. Adrenal/peripheral ACTH is not believed to have significant effects on appetite regulation.

Glucocorticoids (cortisol) are the final product of HPA axis activation during stress. Glucocorticoids are orexigenic, they increase the food intake by acting directly through the central nervous system [34]. In addition to their direct effect, glucocorticoids result in stimulation of orexigenic neuropeptides NPY/AgRP and increase their expression in the hypothalamus [35]. Glucocorticoids also inhibit the release of CRH which is a potent anorectic substance and acts to decrease the NPY/AgRP levels [36]. One significant thing to consider is that glucocorticoids act as orexigenic substance only during non-stress conditions and during the stress, they have a powerful anorexigenic effect. During stressful conditions, glucocorticoids reduce the number and expression of AgRP producing cells [37]. AgRP antagonizes the effect of melanocortins (POMC/ACTH) which act to decrease the food intake [38]. Stress-induced inhibition of AgRP resulting in activation of melanocortin seems to be responsible for the development of stress-induced anorexia.

There are two possible ways in which stress can affect eating. A person exposed to stress either undereats or overeats as stress results in the production of both, orexigenic and anorexigenic substances. This bidirectional relationship between the stress and eating has been demonstrated in various studies. Stress can result in the activation of orexigenic pathways resulting in increased food intake if high calorie/palatable food is available (exact mechanism of this shift is still unknown) [39]. Several factors seem to contribute to this behavior. Chronic stress seems to disrupt sensory-specific satiety (SSS) signaling that generally functions to stop eating the same food again and again [40]. Chronic stress results in the production of glucocorticoids that have potent central orexigenic effects. Alternatively, increased eating may merely be a pleasuring activity that helps counteract the negative feelings associated with stress as food intake has shown to increase calmness and improve mood by decreasing irritability and arousal [41]. This stress-induced hyperphagia can contribute to the development of obesity and highlights the role of stress as a potential factor to cause obesity.

On the other hand, in the absence of high-calorie/palatable foods, stress results in the activation of anorexigenic pathways leading to a decreased food intake [39]. Emotional stress is known to decrease food intake both in humans and animals. Stress can result in activation of pathways that lead to a decreased food intake which can be both acute and sustained over a 24-hour period [42]. A general assumption can be made that acute or repeated restraint stress results in decreased food intake resulting in stress-induced anorexia [43].

Stress also influences the food choices of an individual. Studies have shown that stressed individuals have a preference for nutrient-dense, high-calorie food that is rich in unhealthy sugars and fats [44]. This effect is more prevalent in females than males, and these are the foods that they would typically avoid if they were not stressed [45]. High cortisol reactors (an increase in salivary cortisol from baseline to stress level) tend to consume higher calories when stressed than low cortisol reactors [46]. Men, on the other hand, tend to cope stress with other behaviors such as smoking and alcohol consumption instead of increased eating [47].

## Conclusions

This review seeks to elaborate on the relationship between stress and eating habits by discussing the physiologic regulation of appetite first and then discussing how stress can alter these regulatory mechanisms. There exists a bidirectional relationship between stress and eating. Stress can result in decreased food intake if high-calorie, palatable food is not available. In the presence of high-calorie palatable foods, stress results in increased food intake. This stress-induced hyperphagia can partly be explained by the rewarding and stress-relieving potential of food. Stress can be a potential cause for the development of obesity only if accompanied by increased eating behaviors. This highlights the fact that behavioral modifications can be significant to prevent stress from potentiating the development of obesity.
